# Multiparameter mechanical and morphometric screening of cells

**DOI:** 10.1038/srep37863

**Published:** 2016-12-02

**Authors:** Mahdokht Masaeli, Dewal Gupta, Sean O’Byrne, Henry T. K. Tse, Daniel R. Gossett, Peter Tseng, Andrew S. Utada, Hea-Jin Jung, Stephen Young, Amander T. Clark, Dino Di Carlo

**Affiliations:** 1 Department of Bioengineering, University of California, Los Angeles, CA, USA; 2 California NanoSystems Institute, Los Angeles, CA, USA; 3 Division of Cardiovascular Medicine, Stanford University School of Medicine, Stanford, CA, USA; 4 CytoVale Inc, South San Francisco, CA, USA; 5 Molecular Biology Institute, University of California, Los Angeles, CA, USA; 6 Department of Medicine, University of California, Los Angeles, USA; 7 Department of Human Genetics, University of California, Los Angeles, CA, USA; 8 Department of Molecular Cell and Developmental Biology, University of California, Los Angeles, CA, USA; 9 The Eli and Edythe Broad Center of Regenerative Medicine and Stem Cell Research, University of California, Los Angeles, CA, USA.

## Abstract

We introduce a label-free method to rapidly phenotype and classify cells purely based on physical properties. We extract 15 biophysical parameters from cells as they deform in a microfluidic stretching flow field via high-speed microscopy and apply machine-learning approaches to discriminate different cell types and states. When employing the full 15 dimensional dataset, the technique robustly classifies individual cells based on their pluripotency, with accuracy above 95%. Rheological and morphological properties of cells while deforming were critical for this classification. We also show the application of this method in accurate classifying cells based on their viability, drug screening and detecting populations of malignant cells in mixed samples. We show that some of the extracted parameters are not linearly independent, and in fact we reach maximum classification accuracy by using only a subset of parameters. However, the informative subsets could vary depending on cell types in the sample. This work shows the utility of an assay purely based on intrinsic biophysical properties of cells to identify changes in cell state. In addition to a label-free alternative to flow cytometry in certain applications, this work, also can provide novel intracellular metrics that would not be feasible with labeled approaches (i.e. flow cytometry).

Intrinsic physical properties of cells that reflect underlying molecular structure are indicators of cell state associated with a number of processes including cancer progression, stem cell differentiation, and drug response^1^,^2^,^3^. Nuclear and cytoplasmic structure or morphology have been one of the main tools for histological detection and classification of cancer. These features include chromatin texture, nuclear shape and cytoplasmic features such as shape and cytoplasmic clearing. Morphology is indicative of cell fate, differentiation, and self-renewal capacity. In addition to the expression of certain cell surface markers, cell morphology has been one of the major parameters for validation of pluripotency of human embryonic stem cell (hESC) and induced pluripotent stem cell (iPSC)^4^,^5^,^6^. Recent studies have identified morphological properties that distinguish different subpopulations in highly heterogeneous cultures of mesenchymal stem cells^7^. Morphology-based assays have also been successful in discovery of unique drugs that act on mammalian cells, filamentous fungi, and yeasts^8^. Observation of pharmacological class–dependent morphological changes in cells has been considered as a complementary strategy for drug discovery^6^. Recent work using morphological screening tools have linked morphology to activity of a subset of genes^9^,^10^. While morphometric measurements provide information on visible cell structures without external probing, internal and optically transparent architectural features can be probed by measuring cell deformation under an applied stress. Cell mechanical stiffness has recently emerged as an indicator of various changes in cells state^11^ including cancer cell function, motility, and invasion capacity^12^,^13^,^14^. One study found human metastatic cancer cells to be more than 70% softer than neighboring benign reactive mesothelial cells^1^. Embryonic stem cells have also been found to be more deformable than differentiated cells using atomic force microscopy and micropipette aspiration^15^,^16^. Assaying both external and internal architectural properties of cells through the combinations of morphological and mechanical signatures is expected to provide label-free and low cost biomarkers of cell type or state.

Although cell morphological and mechanical characteristics can be indicative of cell state in a variety of cellular processes and conditions, the lack of high-throughput and integrated methods to assay single-cell physical properties, especially from fluid samples, has been a major barrier to adoption of these platforms^17^. For instance, morphological properties can be measured by automated microscopy, a process that can image tens of cells per second, while cell mechanical properties have been mainly measured using methods such as atomic force microscopy (AFM), optical stretching, or micropipette aspiration, which are single-cell based and manual approaches (<1 cell/sec)^1^,^15^,^18^,^19^. These approaches do not allow for flow cytometry–like throughputs (>1,000 cells/sec) and intuitive readouts, which allow sampling of rare subpopulations of cells in a reasonable time period. Emerging methods are now able to measure a few mechanical properties from tens to thousands of cells per second^20^,^21^,^22^, however, these techniques have not yet provided a holistic view of a cell in which multiple internal and visible features of cellular architecture are simultaneously probed. Multiparameter measurements are important in identifying rare populations of cells, in which additional parameters and sample size provide increased statistical confidence in sub-classification^23^.

In this study, we perform combined mechanical and morphological phenotyping at rates of >1,000 cells/sec using the deformability cytometry (DC) platform. This microfluidic platform was previously introduced by our group, but was only demonstrated to assay a few parameters. The assay is based on microfluidic hydrodynamic stretching of cells combined with high-speed imaging and automated image analysis (Fig.1 and Fig. S1). Briefly, using inertial focusing, single cells arrive at a junction where they are uniformly stretched while being imaged^21^. Here we implement deformability cytometry as a high-throughput automated tool to assay 15 biophysical properties of cells, including cell size and strain, time-dependent mechanical properties, and morphologies across length scales (SI Fig. 1).

We apply this technique to discriminate pluripotent cells from differentiated cells in order to screen for the presence of pluripotent stem cells in mixed cultures. In addition to screening pure samples of pluripotent and differentiated cells at different time points, hESC cells cultured at 0 and two weeks of differentiation were mixed in varying ratios and analyzed. Importantly, we discovered that the combination of morphological and mechanical properties provides significantly higher accuracy compared to either set alone. We also show the capability of this method to correctly classify live and dead cells in mixed samples, and demonstrate proof-of-principle data of utility for drug discovery. Finally, we apply this assay to mixed populations of leukocytes and malignant cells and show its potential in detecting malignant cells in body fluids^24^. This multiparameter data set of cell biophysical properties enables us to use machine learning and statistical data analysis techniques to accurately classify cells, and provides a new method to characterize cell populations across fields – from cancer biology^24^ to immunology^21^, in a label-free, and cost-effective manner. In this paper, we show that the multiparametric nature of our technique is particularly useful for accurately detecting rare phenotypes in heterogeneous samples, when the ”average cell” properties cannot indicate the variations in cellular state within a population.

## Methods

### Deformability cytometry device

The deformability cytometer is a microfluidic device designed for single-cell analysis of cell mechanical properties (SI Fig. 1a)^21^. Cells in suspension are delivered at high rates to an extensional flow which is used to stretch the cells to high strains (SI Fig. 1b). Functioning in an inertial regime (channel Reynolds number *Re* ~ 100), inertial focusing positions cells precisely before stretching (SI Fig. 9a)^25^,^26^ which ensures a more uniform three-dimensional force on cells of the same size. The Dean number *De* =* Re(D*_*h*_*/2r)*^*1/2*^, (where *r* is the radius of curvature of the curved channels upstream the junction and *D*_*h*_ is the hydrodynamic diameter) of the curved channels is around 15. The ratio between the diameter of cells with diameters within 10–20 μm and minimum channel dimension (30 μm) is between 0.3 and 0.6. Based on a previous study by Di Carlo *et al*., these conditions (*De*, particle and channel size) will result in the majority of cells focusing along the centerline equilibrium positions^25^. Cell viscoelastic properties then determine to what extent a cell deforms. This deformation is continuously imaged using high-speed microscopy (SI Fig. 1c) and automated image analysis is conducted to extract cell biophysical properties after transforming images from a polar to a Cartesian coordinate system (SI video 4). Populations of individual cells are plotted based on these parameters in a color density format (SI Fig. 1d). Finite element simulations show that for a simplified model of the system the force applied to a cell at the junction is on the order of 10^−4^ N (SI Fig. 9d), which is almost three orders of magnitude higher than that applied by conventional methods like AFM or micropipette aspiration^27^. The high force allows for a very short deformation timescale (around 2 μs) and large deformations, resulting in processing of more than 1000 cells per second and probing of deep intracellular structures like the nucleus. For more details refer to supplementary methods.

### hESC cell culture and sample preparation

The biophysical properties of human embryonic stem cells (hESCs) (day 0) were analyzed by deformability cytometry before and after feeder and serum-free non-specific induction to differentiation for up to 12 days. Twelve lines of human ESCs, (UCLA1–12) were maintained in DMEM high glucose with 20% knockout serum replacer supplemented with 20 ng/ml of bFGF, and grown on mitomycin-treated mouse embryo fibroblasts^28^ Culture on 1% gelatin coated dishes without feeder cells in DMEM high glucose with 20% FBS resulted in a gradual differentiation^29^. Single cell suspensions were prepared by 5 min treatment with 0.25% trypsin/EDTA solution followed by detachment from the dish, aspiration, and suspension in culture media. For each condition and each replicate three sets of samples were prepared for (i) live cell flow cytometry analysis, (ii) RT-PCR and (iii) deformability cytometry. For deformability cytometry the cell suspension was prepared immediately prior to the test (<1 hour). Cell suspensions were injected into the device, at a concentration of 200,000 to 500,000 cells/mL, using a syringe pump (Harvard Apparatus PHD 2000) and a glass syringe (Hamilton), at flow rate of 900 μL/min.

For complete methods refer to the supplementary information section.

### Multiparameter analysis

#### Expectation maximization

The expectation maximization process was initialized by k-means clustering. Hierarchical search was used to remove parameters that have the least contribution to clustering first. Briefly, exhaustive search was performed at each iteration to find the parameter that yields the lowest clustering error after being removed. This test was performed for data gathered from individual cell lines at day 0 and after two weeks of differentiation. Clustering error was defined as within class variations over between class variations:


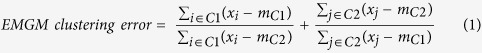


(*x*_*i,*_*x*_*j*_: single data points, *C*1: class number (pluripotent cells), *C*2: class number 2 (differentiated cells). *m*_*C*1,_ *m*_*C*2_: means of classes 1 and 2).

#### Support vector machine and recursive feature elimination

Support vector machine (SVM) with a linear kernel and 5-fold cross validation was used on the pooled data from all 12 hESC lines to test the accuracy of classification. SVMs were implemented using the *SVMTrain* and *SVMclassify* functions of Matlab^TM^. *Perfcurve* function was then used to compute the receiver operating characteristic (ROC) curves for the classifier. The ROC curves are plotted as *True positive rate* or sensitivity defined as: number of true positive instances/(number of true positive instances + number of false negative instances) versus *False positive rate* or 1-specificity, defined as: number of false positive instances/(number of true negative instances + number of false positive instances). Starting with all 15 parameters, the parameters were eliminated hierarchically to minimize misclassification at each iteration. All the data from 12 cell lines at day0 and day14 was used as the training set for SVM and the spiked samples were analyzed for the ratio of single cells belonging to each class.

This study was approved by the Embryonic stem cell research oversight (ESCRO) committee and institutional review board (IRB) of University of California-Los Angeles and the methods were carried out in accordance with the approved guidelines. A written informed consent was obtained from all participants.

## Results

### Gradual changes in deformability and cell size occur upon differentiation

Detailed changes in deformability and size with stem cell differentiation are plotted as 2D single-cell density plots (Fig. 2a, SI Fig. 1d). The median deformability (parameter D3) and median diameter (parameter A) of 12 different human embryonic stem cell lines are plotted before (blue) and after (green) differentiation (Fig. 2c). While pluripotent cell populations are characterized by a higher deformability and smaller diameter, there is a transition to a larger, less deformable state as cells lose their pluripotency upon a 12-day feeder- and serum-free differentiation (Fig. 2a–c, SI Video 1 & 2). The data shows a 15% increase in size and 20% decrease in deformability following two-week differentiation (Fig. 2b). In order to compare deformability to commonly reported elasticity measures, we fabricated agarose beads with elastic moduli spanning 0.2–40 kPa, determined by AFM, and measured their deformability in our device (SI Fig. 2). It is important to note that the AFM measurement is only an estimate of elasticity of the beads in flow, since the elasticity measurements by AFM probe a longer timescale, and measured elasticity may be frequency dependent. Using the standard curve generated from the correlation between AFM and DC measurements of these beads (SI Fig. 2d), pluripotent cells would have a median effective stiffness of ~0.26 kPa which increased to ~1.11 kPa following differentiation. Note that our deformability measurement is not purely elastic, but using the agarose beads, we have shown that we can measure the deformability of particles with a wide range of elastic moduli with this system. Comparisons with cells are made using “effective elasticity”, assuming that the viscosity is matched to that of the hydrogel bead. As both size and deformability of cells change, we used the median value of the ratio between cell deformability and diameter (D3/A) as a simple metric of the relative change of cell stiffness with time (Fig. 2b,d). Normalizing by cell size, which gradually increases from approximately 15 μm to 18 μm upon differentiation (Fig. 2b,e), gives a more accurate representation of changes in cell stiffness due to size-dependent differences in applied stress^21^. Although there are distinctive changes in both size and deformability, there is substantial heterogeneity within a population, such that these two parameters alone only allow for classification accuracy of single cells up to 64.6%.

We assessed pluripotency of cells using conventional pluripotency markers for three of the human embryonic stem cell lines at day 0, 3, 6, 9, and 12 (SI Fig. 3a,b). The results consistently showed down-regulation of pluripotency markers following differentiation as expected. The high correlation (R^2^ ≥ 0.9 for UCLA1 and UCLA2, for the expression of OCT4, NANOG, and TRA-1-81) between our mechanical measure and these commonly used pluripotency markers supports the potential of our method as a label-free assay of pluripotency (SI Fig. 3c).

### Multiple mechanical and morphological parameters improve classification accuracy of single cells

**Figure 1:**
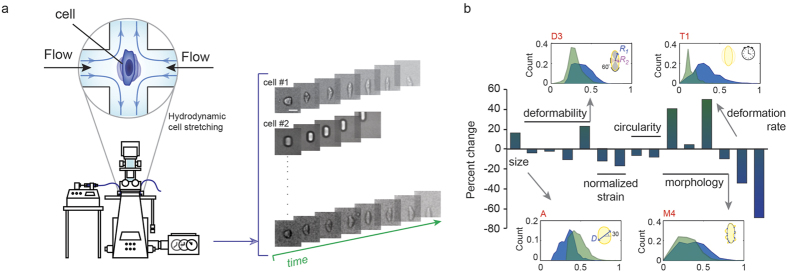
Comprehensive high-throughput quantification of cell physical properties. (**a**) Single cells in suspension are injected into a microfluidic device and are imaged while passing through a hydrodynamic stretching region. Several parameters are extracted from the image series captured from each cell. (**b**) The percent changes in median values of populations of hESCs (blue) and 14 day differentiated hESCs (green) are plotted for 15 parameters extracted from the image series. Histograms of >1000 single cells per condition are shown for select parameters, indicating substantial overlap in population characteristics when only considering a single parameter. (D3: Maximum deformability at the junction, A: Initial cell size, T1: Total deformation time, M4: Morphology metric extracted during deformation defined by the number of intersections of the trace and the moving average or the cell border).

**Figure 2:**
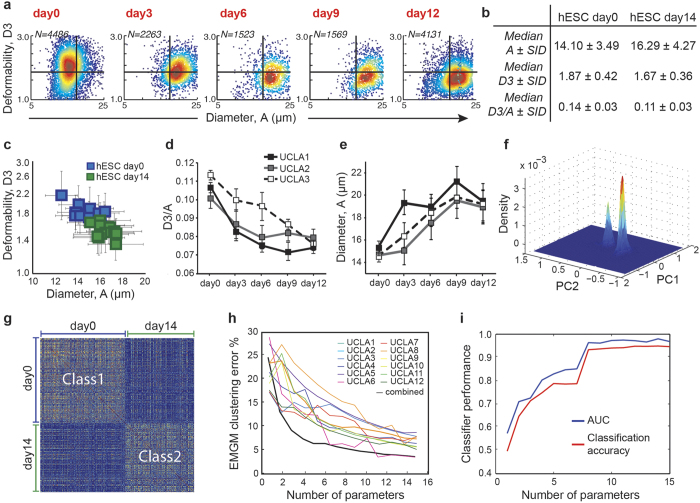
As stem cells differentiate, mechanical properties and pluripotency markers shift (gates are set at Initial Diameter, A =* *17 μm and Deformability, D3 =* *1.6). (**a**) Color density plots of single cell deformability and size measurements of UCLA1 hESCs and hESCs undergoing differentiation for up to 12 days, (red-to-blue indicates high-to-low density of single cell measurements). (**b**) Statistics summarizing the 12 hESC lines are tabulated. The semi-interquartile deviation (SID) value is calculated for median values of the parameters across the 12 cell lines. (**c**) Median deformability and size with SID as error bars are plotted for 12 hESC lines. (**d**) The median value of deformability/diameter (D3/A) gradually decreases (error bars show standard deviation for *n* =* *3 samples per cell line) (plots are shown for three select lines). (**e**) Cells become larger as they differentiate (plots are shown for three select lines). (**f**) The Gaussian kernel density estimation of the most significant principal components (PC1 and PC2) of the data from all 12 cell lines (and all 15 parameters) at day 0 and day 14 shows the existence of two distinct populations in the collapsed multidimensional space. (**g**) Similarity scores comparing the individual data points, calculated by measuring pairwise Euclidean distances between data points, together show the existence of two major blocks of highly similar data points, corresponding to day 0 and day 14 samples. (**h**) Expectation-maximization for Gaussian mixtures (EMGM) clustering for individual cell lines and the pooled data show a decrease in clustering accuracy when parameters are iteratively eliminated. (**i**) Using recursive feature elimination (RFE) and removing parameters one by one, ROC curves for different numbers of parameters show an improvement in classifier performance with larger numbers of parameters.

Beyond cell deformability and diameter, we detected up to a 60% change in the median value of other physical parameters when comparing stem cells and differentiated cell samples, especially in morphology parameters (Fig. 1b). Although differences in the medians between pluripotent and differentiated cell populations were detected, at the single-cell level, we observed substantial heterogeneity, resulting in a significant overlap between the two populations (SI Fig. 4a). Therefore, a more information-rich multidimensional dataset was critical to maximize distances in parameter space between the two cell population clusters. To visualize this improvement in clustering, we compressed the multidimensional dataset into two or three dimensions and observed a marked reduction in overlap at the single-cell level. We first performed principal component analysis (PCA) on the combined 15-dimensional data sets (day 0 and day 14 from all cell lines)—from which, two distinct clusters emerged (Fig. 2f). When we subsequently labeled the groups, we found that two (SI Fig. 4b) or three (SI Fig. 4c) principal components lead to more separated clusters when compared to deformability-size plots alone. These principle components (PCs) consist of linear combinations of our parameters with PC2 and PC3 again being dominated by parameters A, S1, M1, M3, and D4 (parameters with the largest coefficients) (SI Table 2). Although each PC might not be informative of a specific parameter, having a large pool of parameters and using dimensionality reduction are a helpful means for summarizing and visualizing the differences between physical properties of different cell populations. Interestingly, as observed in size and deformability plots, two week–differentiated cell populations appear more heterogeneous than day 0 pluripotent cells, displaying a more spread distribution in PC space (SI Fig. 4b,c). The increased heterogeneity in this population can be better quantified when looking at a similarity matrix for each individual cell, showing a higher similarity between single day 0 cells compared to day 14 cells (Fig. 2g). The similarity matrix is generated by calculating pairwise Euclidean distances between every row of the pooled data set (red showing smaller distances or higher similarity). The existence of two distinct clusters in the data is also visible from the similarity matrix.

To determine the optimal number of parameters for successful clustering of the two classes (pluripotent versus differentiated cells), we performed unsupervised clustering by expectation maximization for Gaussian mixture (EMGM) models considering different numbers of parameters. As expected, increasing the number of parameters resulted in increased clustering accuracy across the 12 cell lines separately (Fig. 2h, colored lines). Similarly, pooled data from all cell lines at day 0 and day 14 yielded increased clustering accuracy with increasing the number of parameters (Fig. 2h, black line). Although adding more pieces of information enables more accurate clustering, increasing the number of parameters from 9 to 15 parameters resulted in only a 2.35% reduction in clustering error, compared to a 20.2% reduction from 2 to 9 parameters. This could be due to the fact that not all the features extracted from the images are linearly independent. In fact, the correlation coefficients between parameters show partial dependence between a few parameters as expected (SI Table 3).

Using these same 9 most important features, we next trained a support vector machine (SVM) classifier to distinguish between single pluripotent and differentiated cells with 93% classification accuracy. When increasing to 15 parameters, classification accuracy increased only to 95%. A 5-fold cross validation technique was first used to train the classifier and classification accuracy was calculated as the fraction of data points that were classified correctly. The area under the curve (AUC) of the sensitivity *vs*. specificity receiver operating characteristic (ROC) was ~0.97 using all 15 parameters, showing an exceptional classifier performance (SI Fig. 4d). SVM recursive feature elimination (RFE) was then used to determine whether a smaller number of parameters could yield high classification accuracy as well. The parameters were removed one at a time depending on their contribution to classification, i.e. the parameters for which their removal resulted in the least loss of accuracy were removed first (SI Fig. 4e). Again, we observed a significant decrease in classification accuracy and AUC upon removing 6 or more parameters (Fig. 2i) or when only applying parameters extracted prior to junction stretching (SI Fig. 5).

### Nuclear and cytoskeletal contributions to biophysical parameters

**Figure 3:**
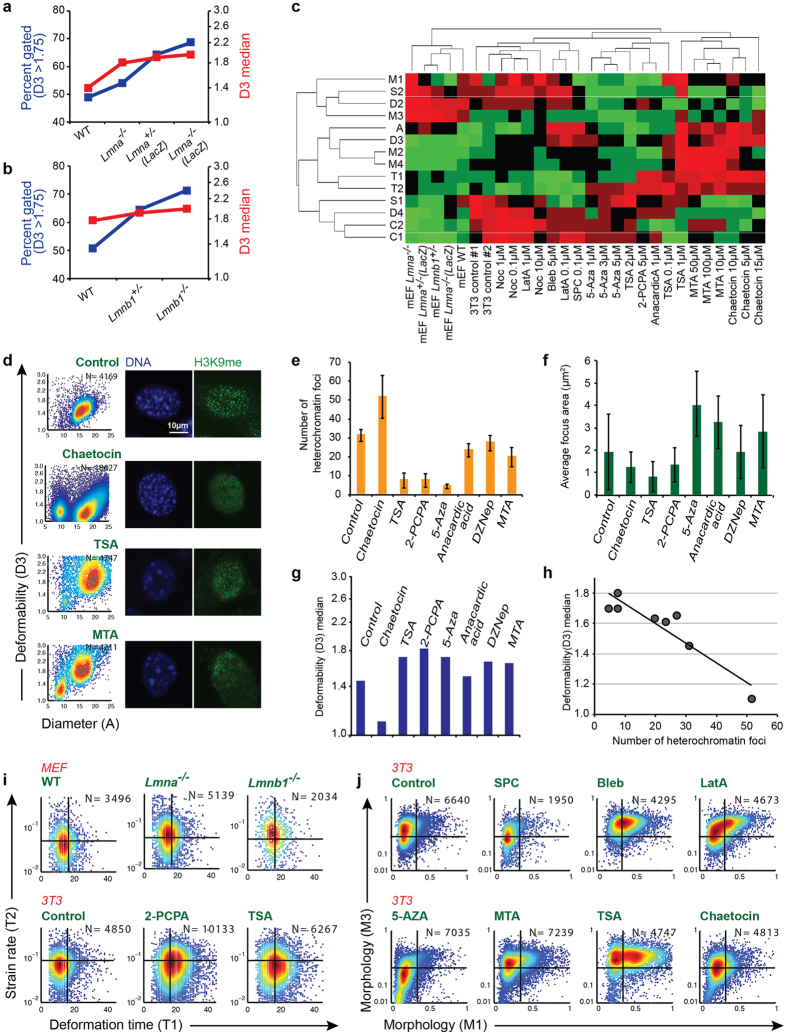
Nuclear and cytoskeletal origins of measured biophysical characteristics. (**a**) Mouse embryonic fibroblasts (MEFs) lacking A-type lamins are more deformable than wild-type MEFs. *Lmna*^−/−^ (*LacZ*) cells were more deformable compared to heterozygous samples *Lmna*^+/−^ (*LacZ*), which are more deformable than *Lmna*^−/−^ cells. (**b**) Lamin B1 knockout cells (*Lmnb1*^−/−^) are softer than heterozygous and wild-type MEFs. (**c**) Using mechanical and morphological properties of the 3T3 and embryonic fibroblast cells, linear discriminant analysis (LDA) was used to group the cells. Cells that were treated with multiple chromatin modifying drugs, cells with knocked out lamins and cells treated with several drugs that reorganize cell cytoskeleton are grouped separately. (**d**) NIH-3T3 fibroblasts exposed to chromatin reorganizing drugs were observed to have variations in chromatin condensation and numbers of heterochromatin foci (bright signal) by confocal imaging of Hoescht stained nuclei (blue). H3K9 methylation immnuostaining is also shown (green). (**e**) The number of heterochromatin foci as a measure of chromatin condensation level is shown for 3T3s after treatment with chromatin-modifying drugs. (**f**) Average heterochromatin focus area is shown in each case. (**g**) Cell deformability also changes as a result of exposure to these drugs. (**h**) Cell deformability inversely correlates with the number of heterochromatin foci (*R*^*2*^ =* *0.91). (**i**) While strain rate (Up to 40% increase in lamin knockout cells compared to wildtype cells) and deformation time (up to 70% increase in chromatin-reorganized cells compared to control) were the two parameters found to best discriminate cells with modified nuclear structure, (**j**) changes induced by cytoskeletal drugs were best distinguished by their morphological properties (Up to 2X change in morphology parameters).

We studied the effect of nuclear lamins, chromatin state, and cytoskeletal structure to evaluate which molecular changes most affect mechanical readouts extracted from DC measurements. Using DC, we previously did not observe any significant change in cell deformability upon treatment of HeLa cells with cytoskeletal modifying drugs including Latrunculin A to disrupt actin polymerization and nocodazole to inhibit microtubules^21^. However, all *Lmna* knockout mouse embryonic fibroblasts (MEFs) showed slightly higher deformability compared to the wild-type cells, and knockout of *Lmna* by insertion of *LacZ* (*Lmna*^−/−^ (*LacZ*)) resulted in larger deformation compared to heterozygous knockout (*Lmna*^+/−^ (*LacZ*)) Additionally, *Lmna*^−/−^ cells were stiffer than both *Lmna*^+/−^ (*LacZ*) and *Lmna*^−/−^ (*LacZ*) (Fig. 3a). Unlike *Lmna*^−/−^ cells, knockout by *lacZ* insertion is expected not to express truncated form of lamin A^30^,^31^. We also detected greater deformability in *Lmnb1*^−/−^ cells compared to wild-type MEFs (Fig. 3b). *Lmnb1*^−/−^ cells were more deformable than heterozygous cells (*Lmnb1*^+/−)^. In addition to deformability, we detected an increase in parameter T2, the strain rate, for *Lmna*^−/−^ (*LacZ*) and *Lmnb1*^−/−^ cells in our device (Fig. 3i).

Next, to study the effect of chromatin structure on measured biophysical properties, we treated 3T3 fibroblasts and Jurkat acute T-cell leukemia cells with several chromatin reorganizing drugs (Fig. 3d, SI Fig. 6). Hoechst 33258 dsDNA staining was used to visualize nuclear reorganization after treatment with chromatin modifying drugs (Fig. 3d). The number of heterochromatin foci was used as an indicator of chromatin condensation level^32^. We quantitatively evaluated the number of heterochromatin foci and average area of the foci in at least 20 cells per sample (Fig. 3e,f). We found that deformability is inversely correlated with the level of chromatin condensation (Fig. 3g,h). Cells that were treated with the histone deacetylation (HDAC) inhibitor, TSA and DNA methyltransferase (DNMT) inhibitor, MTA and DZNep, appeared to have a less condensed chromatin by microscopic analysis. These cells were more deformable (more than 20% increase in the average deformability of the population) compared to untreated 3T3s. 3T3s and Jurkats treated with the H3K9 methylation inhibitor, Chaetocin, on the other hand, were less deformable and had more condensed chromatin. We did not detect any significant difference in the deformability of DNMT triple knockout mouse embryonic stem cells compared to wild-type cells (SI Fig. 7). Perhaps, in constitutive knockouts, other adaptive responses may be occurring over the longer time periods. Alternatively, it is possible that DNA methylation alone does not contribute a major biophysical property to mouse ESCs. Contrary to mouse ES cells, we observed an increase in deformability of somatic cells in response to MTA and we know that somatic cells cannot tolerate loss of methylation. One explanation could be that DNA methylation does have a major role in biophysical properties of somatic cells but not in a pluripotent state. Alternatively, removing methylation by MTA may lead to loss of histone methylation as well and these two together may lead to increased deformability. We also observed a significant increase in deformation time (parameter T1) in cells treated with TSA and 2-PCPA, compared to non-treated cells (Fig. 3i, bottom row). As it has been previously hypothesized, this could be partly reflecting a change in nuclear plasticity upon chromatin reorganization^15^. *Lmna*^−/−^ cells, however, did not show any significant change in deformation time (Fig. 3i, top row) compared to wild-type cells. Significant changes in cell morphology were also observed upon treatment with several of the chromatin-modifying drugs (Fig. 3j). Treatment with cytoskeletal drugs, although not significantly affecting deformability, led to significant changes in cell morphology features (Fig. 3j).

Unique multiparameter biophysical profiles are drug dependent. Using Linear Discriminant Analysis (LDA), while mouse embryonic fibroblasts (wild-type and lamin knockout) grouped as a separate cluster, in a separate cluster of 3T3s, non-treated and cytoskeletal drug–treated cells were separated from chromatin-modifying drug–treated cells (Fig. 3c). Using this phenotypic analysis, a drug with unknown mechanism of action could potentially be classified based on its effect on the biophysical phenotype of cells under treatment and how it clusters with cells treated with previously known drugs.

### Impurities in mixed population samples can be detected using biophysical properties

By gathering multidimensional data from separate samples belonging to each sub-population of a mixed-population sample as the training set, we can build a support vector machine (SVM) classifier to provide a classification boundary for unknown samples. These samples ideally are collected under independent conditions to increase the generalizability of the classification. We tested these classifiers in separate samples in which we spiked cells: (i) to distinguish pluripotent and differentiated cells in partially differentiated samples of hESCs in order to screen for the presence of pluripotent stem cells in mixed cultures, (ii) to quantify the cytotoxicity of an HDACi (histone deacetylase inhibitor), Trichostatin A (TSA) over longer time periods, and (iii) to detect malignant cells in mixed samples of white blood cells.

#### i) Classifying pluripotent and differentiated cells

**Figure 4:**
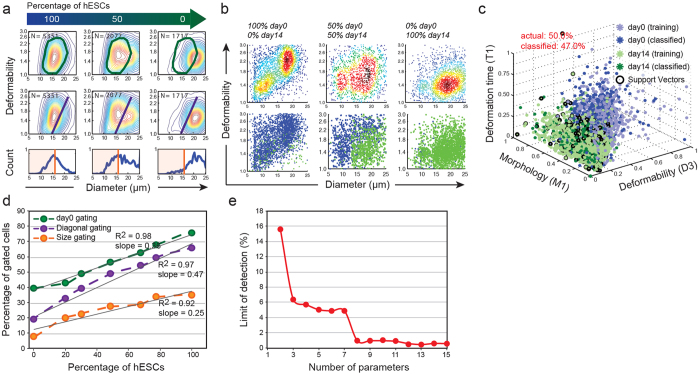
(**a**) Three gating methods were applied to mixed samples: “Diagonal gating”, “day 0 gating”, and “size gating”. (**b**) Using all 15 parameters, single cells were accurately classified as day 0 (blue) or day 14 (green). (**c**) Cells classified as day 0 and day 14 are shown in the sub-space of 3 parameters: D3, M1, and T1. Data from all 12 cell lines for pure day 0 (light blue) and day 14 (light green) were used as training sets. (**d**) Percentage of gated cells versus percentage of spiked day 0 cells. The slope of the curves shows the sensitivity of prediction to variations in spiked ratios. The lower the slope the larger the errors caused by sample-to-sample variations and when deviating from a 1:1 spiking ratio. (**e**) Increasing the number of parameters enables the distinction of lower impurities in samples. The “Limit of detection” was calculated as the inverse of analytical sensitivity, where analytical sensitivity is the normalized slope of the curve (normalized by the maximum standard deviation (*n* =* *3 sets of samples) of the percent cells classified as day 0, across different mixing ratios.)

We observed that a combination of biophysical parameters is important for high accuracy classification of mixed pluripotent and differentiated cell samples with SVM using a Gaussian kernel (Fig. 4e). For comparison, three flow cytometry–like gating strategies were first applied to assess the purity of hESC cells in mixed samples using only deformability and initial cell diameter (parameters D3 and A) (Fig. 4a). “*Size gating” was performed by selecting cells with initial diameter (parameter A) smaller than 15 μm. “Diagonal gating” selects cells with deformability (parameter D3) larger than 1.4 and diameter (parameter A) smaller than 15 μm. “Day 0 gating” was applied such that it would contain 75% of the population of 100% day0 sample.* While all three methods correlate well with the actual purity, “*Diagonal gating*” and “*day 0 gating*” are more sensitive to impurities achieving a slope closer to an ideal 1 (Fig. 4d). In comparison to simple gating, machine learning–based classification with additional parameters lead to improved detection of spiked sub-populations (Fig. 4b, second row, SI Fig. 8: cells classified as pluripotent in blue and differentiated in green). Using all 15 parameters, we can detect as small as a 0.7% impurity difference across samples (Fig. 4e). Support vectors clearly define a boundary when visualizing the data in 3D with test data (spiked cells) overlapping well with the training data from all 12 cell lines at day 0 - Class 1 and day 14 - Class 2 for the 5 different spiking ratios (Fig. 4c). Upon removing parameters using recursive feature elimination (RFE), the sensitivity of our sub-population estimation decreased and the minimum distinguishable sample impurity increased (Fig. 4e). Using RFE, deformability (D3), size (A), deformation time (T1), and morphology (M1) were the most important parameters contributing to accurate classification.

#### ii) Application as a viability (cytotoxicity) assay

**Figure 5:**
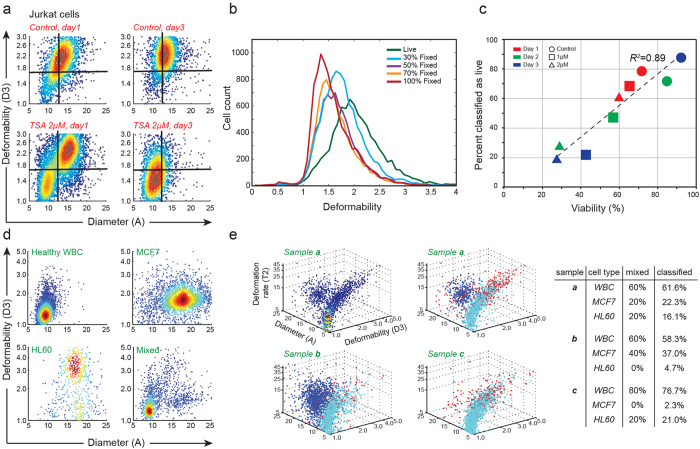
(**a**) Density profiles of Jurkat cells after 1–3 days of treatment with TSA (1, 2 μM) show that at higher dosages and treatment times, a portion of the population becomes stiff and small. (**b**) The profile of spiked and pure samples of live and fixed Jurkat cells. (**c**) Data shows high correlation between predicted viability and viability calculated using a standard cytotoxicity assay. (**d**) 2D profiles for healthy leukocytes (WBC), MCF7, and HL60 cells. (**e**) Three-dimensional profiles of diameter (A), deformability (D3), and deformation rate (T2) for three differently mixed samples of WBCs (cyan), HL60s (red), and MCF7s (blue). Classification using all 15 parameters led to < 5% error in classification of three differently mixed samples.

We had previously observed that while cell size remains constant, fixation with 4% paraformaldehyde increases cell stiffness as expected. Spiked samples of live and fixed Jurkat cells at different percentages show a decrease in the median deformability of the mixed populations with larger percentage of fixed cells (Fig. 5b). Although experimentally treating cells with paraformaldehyde is a controlled way to decrease the deformation of cells, it is not a representative of natural cell death, or induced, apoptotic death. Therefore, we next used Jurkat cells treated with trichostatin A (TSA). TSA is currently being researched into its role as a cancer therapy since it often results in expression of apoptosis inducing/regulating genes, which are normally turned off in cancerous cells. Jurkats were exposed to two different doses of TSA (1 μM and 2 μM), and deformability changes were observed for 3 continuous days of treatment, longer time periods than when observing the effect on chromatin previously. There was an initial increase in deformability as discussed above, however, at later time points, size and deformability decreased again (Fig. 5a). After a single day, two unique sub-groups emerged in samples treated with 2 μM TSA. One sub-group had a slightly increased mean size and deformability, whereas the smaller sub-group had a highly decreased deformability and size. However, by day 3, there was a noticeable trend of cells becoming smaller and less deformable – merging into the smaller subgroup. Increased cells classified in this group using multiparameter SVM correlated with decreased cell viability (Fig. 5c).

#### (iii) Identifying malignant cells in body fluid samples

We have previously observed unique signatures from cancer cells disseminated in pleural effusions even when considering only size (A) and deformability (D3)^24^. Here, we built the training set for multiparameter biophysical analysis of potential cells within pleural effusions using samples of healthy white blood cells, MCF7 breast cancer cells, and HL60 leukemia cells (Fig. 5d). Building a SVM classifier and using RFE, diameter (A), deformability (D3), and deformation rate (T2) were found to be the three most important parameters. To evaluate the accuracy of our method in distinguishing different populations, we then mixed WBCs, HL60s, and MCF7s at different ratios and used the previously trained SVM classifier to predict the three cell types. We detected less than a 5% difference between the classifier predictions and the actual ratio of mixing in the three spiked populations tested (Fig. 5e, right). Note that, although these cells can be quantified and distinguished using flow cytometry and other label-based methods, our approach is purely physical, does not require labels, or the associated sample preparation.

## Discussion

Here, we introduced a high-throughput label-free approach for classifying cells suspended in solution purely based on their physical properties. Using image analysis followed by machine learning algorithms, the multidimensional data captured from high-speed images of deforming cells can be used to classify target cells for several applications. Comsol simulations show that for a simplified model of the system, the force applied to a cell at the junction is on the order of 10^−4^ N (SI Fig. 9), which is almost three orders of magnitude higher than that applied by conventional methods like AFM or micropipette aspiration^27^. The high force allows for a very short deformation timescale (around 2 μs), resulting in processing of more than 1000 cells per second.

Chromatin and nuclear membrane elasticity are expected to be dominant contributors to deformability metrics because of the large stresses that we can apply which result in corresponding large strains to the whole cell and nucleus. Our data supports a dominant role for nuclear architecture in our deformability measures. Besides the high deformability of ESCs compared to differentiated cells, results with chromatin modifying drugs directly link the heterochromatin abundance and deformability for two cell types. Our dynamic measurements of deformation at high shear rates are also expected to report on complex mechanical impedance and time-dependent deformation characteristics of the cell, which can reflect the ability of chromatin to flow within the nucleus, in which open euchromatin regions are expected to lead to an increased ability to stretch with the extensional flow and remain stable in the junction. This is supported by data linking increases in deformation time measure (T1) with chromatin-modifying drugs. Chromatin rearrangements and lamin expression, which are critical for cell development and differentiation, are also involved in many cellular processes, human conditions, and diseases including aging, cancer, and cardiovascular disease^33^,^34^,^35^. Note that the direct relationship between cell viscous properties and the time it takes for the cell to deform (T1) has not been defined here. T1 is a dynamic parameter that partly reflects cell plasticity.

Such a label-free approach may find use in the field of regenerative medicine, since heterogeneity in culture systems is a challenge for repeatability. Furthermore, assessment of pluripotency in human stem cells currently requires teratoma formation in mice. This is not a quantifiable assay, and merely provides a binary yes–no result with significant time investment and cost. A simple label-free method to evaluate cultures for self-renewal potential or level of differentiation has the potential to enable low cost quality control (QC) of stem cells with robust quantifiable measurements. Experiments correlating teratoma formation from a cell batch with deformability profiles would be a necessary next step. It is also possible that these types of measurements may ultimately enable a diagnostic for differentiation potential in future studies. In particular, based on the machine learning approaches presented here, we can create a classifier that identifies normal pluripotent cultures that yield the best differentiation potential. One could then screen for cultures that fall outside of this validated state.

Beyond differentiation, our technique can be used to measure other cell states, particularly when nuclear architectural changes are prevalent. Reorganization of cell cytoskeleton and nucleus has been observed in cells when exposed to cytotoxic agents, such as chemotherapies, that lead to cell death^36^,^37^,^38^. These reorganizations result in changes in cell mechanical properties^39^,^40^. For example, Paclitaxol is a chemotherapeutic drug that is used for the treatment of breast and ovarian cancer. It acts by interfering with the normal function of microtubules and inducing apoptosis in cells^41^. Significant morphological and shear modulus changes has been observed in cells treated with Paclitaxol^42^. Therefore, changes in cell biophysical properties could act as an indicator of treatment response and drug cytotoxicity.

Aberrant epigenetic regulation, causing undesirable gene silencing or expression, has also been observed frequently in cancer, and the global level of chromatin condensation controlled by these epigenetic modifications is found to considerably alter the gross structural and presumably physical properties of the cell nucleus. In fact, nuclear shape and structure are still one of the main tools for histological detection and classification of cancer^43^. However, current cytomorphological analysis is labor-intensive and qualitative, creating a need for automated, quantitative alternatives^24^. Using purely physical properties of cells, we showed accurate detection of malignant cell sub-populations in multiple mixed samples. Identification and classification of subpopulations of primary cells is another important step to show the clinical utility of this technique. Applying multiparameter classification to identify disseminated malignant cells in body fluids such as pleural effusions, ascites, and urine may further enhance diagnostic performance^24^. The additional mechanical and morphological properties we can assay here are poised to accelerate the development of physical biomarkers across a range of fields, particularly impacting immunology and cancer biology, in which whole-cell architectural changes are critical aspects of disease processes.

One application for using the high-dimensional mechanical phenotyping approach is identifying unknown subpopulations in a heterogeneous sample or an unknown effect on a sample. The aim here is to identify the number of clusters with unique biophysical signatures and unique properties within each cluster. This can be achieved using unsupervised clustering algorithms after quantifying single cell properties with the deformability cytometry platform. Forming similarity matrices of the data gathered from single cells is one such approach. The similarity matrix is formed by calculating the pairwise Euclidean distances between the rows of the pooled data. The existence of distinct blocks would be indicative of different subpopulations. Another way of identifying different subpopulations in an unknown sample is using principal component analysis (PCA). Drug discovery is another application, where instead of classifying subpopulations of cells, classification of the drug effect on the cell population is of interest. For this case the training data set can be collected by gathering data from one specific cell line treated with a number of different drugs known to have different mechanisms of action. The number of different classes (e.g. mechanisms of action) could also be inputted into the classifier. For supervised learning, the size of training data depends on the heterogeneity of the cell line response, the acceptable error margin and the selected classifier. In the case of day0 versus day14 hESCs, using all the features and a linear kernel, we selected different numbers of data points as our training set and plotted the training error vs. the number of training samples. We observed that beyond 4000–5000 training data points our misclassification error did not decrease significantly. For specific applications it would be beneficial to collect an initial set of samples to plot learning curves and train the model and make sure the model is not over- or under-fitting the data.

Methods to sort out different cell sub-populations are also needed. Currently, sorting can be done using traditional fluorescence-activated cell sorting techniques; however, the addition of antibody labels to cell surface proteins and the required sample preparation have a substantial associated regulatory burden that would be a roadblock to using these approaches for clinical products. Although we do not currently demonstrate sorting in our system, we anticipate that this functionality will be achievable in the future using hardware-based image analysis^44^ and traditional flow cytometry sorting hardware.

## Additional Information

**How to cite this article**: Masaeli, M. *et al*. Multiparameter mechanical and morphometric screening of cells. *Sci. Rep.* **6**, 37863; doi: 10.1038/srep37863 (2016).

**Publisher's note:** Springer Nature remains neutral with regard to jurisdictional claims in published maps and institutional affiliations.

## Supplementary Material

Supplementary Video 1

Supplementary Video 2

Supplementary Video 3

Supplementary Video 4

Supplementary Information
