# Supplementary material for: Multiparameter mechanical and morphometric screening of cells
Source: Sci Rep. 2016 Dec 2;6:37863. doi: 10.1038/srep37863 (PMC5133672; doi:10.1038/srep37863)
Supplement: Supplementary Information [file srep37863-s5.doc]

**Multiparameter mechanical and morphometric screening of cells**

Mahdokht Masaeli^1,2,3^, Dewal Gupta^1^, Sean O’Byrne^1,2^, Henry T.K. Tse^1,2,4^, Daniel R. Gossett^1,2,4^, Peter Tseng^1,2^, Andrew S. Utada^1,2^, Hea-Jin Jung^5^, Stephen Young^5, 6, 7^, Amander T. Clark^8,9^, Dino Di Carlo^1,2^

1. Department of Bioengineering, University of California, Los Angeles, CA. 2. California NanoSystems Institute, Los Angeles, CA. 3. Division of Cardiovascular Medicine, Stanford University School of Medicine, Stanford, CA. 4. CytoVale Inc, South San Francisco, CA. 5. Molecular Biology Institute, University of California, Los Angeles, CA. 6. Department of Medicine, University of California, Los Angeles, USA. 7. Department of Human Genetics, University of California, Los Angeles, CA. 8. Department of Molecular Cell and Developmental Biology, University of California, Los Angeles, CA. 9. The Eli and Edythe Broad Center of Regenerative Medicine and Stem Cell Research, University of California, Los Angeles, CA.

**SUPPLEMENTARY INFORMATION**

**SUPPLEMENTARY METHODS**

**Microfluidic device fabrication.**

Microfluidic devices were fabricated using common polydimethylsiloxane (PDMS) replica molding processes. Briefly, standard lithographic techniques were used to produce a mold from a silicon wafer spin-coated with SU-8 photoresist. PDMS chips were produced from this mold using Sylgard 184 Elastomer Kit (Dow Corning Corporation) and a cross-linker to polymer ratio of 1:10. To enclose the channels, PDMS and glass were both activated by air plasma (Plasma Cleaner, Harrick Plasma, 500 mTorr, 30 sec) before being bonded together. The device contained 20µm filters to avoid entrance of cell clusters or dust followed by curving channels to ensure inertial focusing and a junction that provides an extensional flow. Channel width before and after extensional region was 67µm and the height of the channel was 30µm. The positioned cells arrived one at a time at an extensional flow, were stretched and left the junction from either of the two outlets on the top or bottom. The extensional region was continuously imaged using high-speed microscopic imaging.

**Drug testing experiments.** We treated NIH-3T3 fibroblasts and Jurkat acute T-cell leukemia cells with several cytoskeletal drugs to: inhibit microtubules with nocodazole (0.1,1 μM), inhibit nonmuscle myosin II with blebbistatin (5 μM), disrupt actin polymerization with Latrunculin A (0.01,1,10 μM) and modify Keratin network architecture by sphingosylphosphorylcholine (SPC) (0.1 μM) for two hours and chromatin modifying drugs : Chaetocin, an inhibitor of H3K9 methylation (5-15 µM), Trichostatin A (TSA), an inhibitor of histone deacetylation (0.1-2µM), 5’-Deoxy-5’-(methylthio)adenosine (MTA), (10-100 µM) and 5-Azacytidine (5Aza), (1-5µM) , inhibitors of DNA methylation and 3-deazaneplanocin A (DZNep), an inhibitor of S-adenosylmethionine-dependent methyltransferase (10µM) for 48 hours. Hoechst 33258 dsDNA staining was used to visualize nuclear reorganization after treatment with chromatin modifying drugs. The number of heterochromatin foci was used as an indicator of chromatin condensation level.

**Leukocyte and cancer cell preparation and spiking.** WBC samples were obtained from healthy donors and red blood cells were removed by hypotonic lysis with Red Blood Cell Lysis Buffer as prescribed (Roche Applied Science). Written informed consent was obtained from each individual. The remaining cells were resuspended in DMEM and were incubated at room temperature for 20 minutes. The MCF7 cell line (ATCC HTB -22) was maintained in DMEM -F12 with 0.01 mg/mL bovine insulin and 10% (vol/vol) fetal bovine serum. Cells were washed with phosphate-buffered saline (PBS) and dissociated with 0.25% trypsin/EDTA solution into single cell suspension. The HL60 cell line (ATCC® CCL-240™) was cultured in RPMI-1640 Medium supplemented with 10% Fetal Bovine Serum. Mixed cell suspension samples at 6 different ratios of WBC:MCF7:HL60 were prepared for the assay: (100:0:0, 0:100:0, 0:0:100, 60:20:20, 60:40:0, 80:0:20)

**NIH 3T3 and Jurkat cell culture.** NIH 3T3 cell line was maintained in DMEM-F12 with 10%(vol/vol) fetal bovine serum (FBS) and 1%(vol/vol) Penicilin/Streptomyci. Jurkat cell line was maintained in RPMI with 5%(vol/vol) FBS.

**Lamin and DNMT knockout cell lines.** Primary mouse embryonic fibroblasts (MEFs) were isolated from *Lmna*−/− ^1^, *Lmnb1*−/−^2^, *Lmnb1*−/−^3^, and wild-type littermate mouse embryos at E13.5. After removing the head, liver, heart, and other viscera, the remainder of the embryo was minced with a razor blade and incubated with 0.25% trypsin-EDTA (Gibco) at room temperature for 20 min, followed by repeated pipetting to make a single cell suspension. All fibroblasts were cultured in DMEM medium containing 10% FBS in 5% CO_2_ at 37°C. Dnmt1−/− Dnmt3a−/− Dnmt3b−/− (TKO) mouse ES cells and control cells were provided by Masaki Okano RIKEN Center for Developmental Biology ^4^. Loss of DNMTs in TKO cells was confirmed by both genotyping and resistance to antibiotics engineered into the mutant allele.

**Fixation experiments.** Jurkat acute T-cell leukemia cells were fixed in 4% paraformaldehyde for 20 minutes and mixed with live Jurkat cells at different ratios (0, 30, 50, 70 and 100 percent fixed cells). Physical properties of single cells in these mixed samples were then assessed using our deformability cytometry device.

**Long-term TSA treatment and cell viability determination.** Jurkat cells were treated with 1µM and 2µM Trichostatin A (TSA) for up to 3 days. Following incubation with TSA at different concentrations and time points, cell samples were split in half; one half was analyzed using our setup. Traditional viability assay was used to assess the viability of the other half of the samples. Briefly, cells were washed with serum-free DMEM and incubated with 4mM Ethidium Homodimer-1 (EthD-1) and 2mM CalceinAM for 30 minutes at 37°C. Cells were then imaged using fluorescence microscopy and viability ratio was quantified using ImageJ. We trained the SVM classifier using Day 0 cells with no TSA treatment as our “Live” class and cells treated with 2µM TSA after 3 days as the “Dead” class. EthD-1 and Calcein AM were obtained from Invitrogen (Carlsbad, CA).

**High-speed imaging and data extraction.** Image sequences from cells at the extensional flow region magnified with a 10× objective (Nikon Japan 10x/0.30 on a Nikon Eclipse Ti inverted microscope) were recorded at ~520,000 frames/s using a Phantom v7.11 high-speed camera and the Phantom Camera Control Software (Vision Research Inc.). Automated image analysis was conducted on the gathered cell deformation images to track cells, and to extract 15 parameters based on cell biophysical properties (SI Video 3). The cell tracking and analysis software was previously developed in our lab ^5^. The custom image analysis script performs image processing and data collection on each 1.5 second experiment totaling 780,000 frames, with expected occurrences of 1,000 to 5,000 cells.

The following parameters were extracted from the captured high-speed images:

(A) Initial cell size (µm), before reaching the junction by calculating the maximum diameter of the cell within 30 degrees from the horizontal axis.

(D1) Deformation of cells at the junction, accounting for pre-junction perceived deformations due to morphology.

(D2) Deformation of cells at the junction defined as: (long axis-short axis) / (long axis+short axis)

(D3) Maximum deformability at the junction defined as the maximum cell diameter within 30 degrees from the vertical axis divided by the minimum diameter within 30 degrees from the horizontal axis.

(D4) The maximum vertical diameter at the junction (µm).

(S1) Maximum deformability at the junction normalized by cell perimeter before deformation.

(S2) Relative strain defined as the ratio of parameters D3 and A (µm^-1^).

(C1) Circularity defined as cell perimeter/area ratio at the maximum deformation (µm^-1^).

(C2) Circularity defined as the perimeter/area ratio prior to deformation in the channel (µm^-1^).

(M1) Morphology extracted prior to the junction measured by the difference between the area under the curve of the cell border (trace) and its moving average. This parameter indicates cell roughness (AU). All morphology parameters are normalized to the maximum value detected for the cells under this study.

(M2) Morphology metric extracted prior to deformation as defined by the number of intersections of the trace and the moving average (AU). This parameter indicates regularity of the surface.

(M3) Morphology metric extracted during deformation is defined as in M1 (AU).

(M4) Morphology metric extracted during deformation is defined as in M2 (AU).

(T1) Length of deformation time between when the cell enters the junction until it leaves (µs).

(T2) The rate of change in deformation at the junction defined by change in vertical cell diameter over the first 5 frames of the deformation (m/s).

**Numerical simulations and force approximation.** To estimate fluid pressure and the force applied on cells at the junction, we simulated steady-state pressure and force using a numerical model that solves the 3D incompressible Navier-Stokes equations. To achieve this, we assumed that the position of the cell is ﬁxed at the center of the junction and the cell is not deforming or rotating in time. Using this method, we conducted a series of simulations for various inlet flow rates specified as input velocity boundary conditions.

**Agarose bead fabrication.** A microfluidic droplet generator platform was used to generate agarose beads with different elastic moduli ^6^ (SI Fig. 2b). The pre-gel solution was prepared by melting and diluting two low-gelling temperature agarose types: Agarose Type IX and IXA (Sigma) at different dilutions (0.5, 1 and 2 wt%) in DI water. The oil phase consisted of mineral oil (Sigma) mixed with 2% Span80 (Sigma). The bead generation process was performed on an incubated microscope (at 40°C) to prevent agarose gelation. After capturing beads (10-20μm in diameter), the solution containing oil and surfactant was incubated at 4°C overnight to allow the gelation of agarose droplets. The beads were then resuspended in DI water for analysis.

**Elastic modulus characterization:**Elastic moduli of the two agarose gel types were measured in bulk at different dilutions. Force-displacement curves were obtained from a Bruker Catalyst AFM aligned above a Leica inverted microscope. Model SNL-D cantilevers (Bruker, spring constant: 0.06 N/m) generated indentation curves in force control mode. Elastic modulus was extracted by fitting the Hertz model to our acquired data. Here, we are interested in understanding the range of operation of the deformability cytometry system rather than assigning an elastic modulus to the deformability measurement. Although not being exactly equal, bulk measurement of the gels is representative of relative elastic moduli of different beads.

**Immunofluorescence microscopy.** Human embryonic stem cells and mouse 3T3 fibroblasts were fixed in 4% paraformaldehyde (Fisher Scientific, US) in phosphate-buffered saline (PBS, Invitrogen) at room temperature for 20 minutes. Cells were then washed three times in PBS and then permeabilized in 0.1% Triton X-100 in PBS for 10 minutes. Cells were washed three times again and incubated with goat anti-Lamin A/C (Santa Cruz) diluted 1:400 and rabbit anti-Histone H3K9me1me2me3 (Active Motif) diluted 1:500 in 1%BSA for 1 hour at room temperature. Cells were washed three times in PBS and then incubated with a 1:400 dilution of Cy5-labeled chicken anti goat and 1:400 FITC-labeled mouse anti-rabbit and 1:10000 Hoechst 33342 (Invitrogen) for one hour. The cells were then washed one last time in PBS and were mounted in Prolong antifade mounting medium (Invitrogen) on glass slides for confocal imaging.

**Flow Cytometry.** Expression of cell surface pluripotency antigens was characterized using flow cytometry. After harvesting cells by trypsin-EDTA, cells were washed in cold 1% BSA in PBS, were passed through a cell strainer (40μm Nylon membrane BD Falcon) and resuspended to approximately 10^6^ cells/mL of cold BSA/PBS solution. Cell samples were incubated with optimal dilution of DAPI, PE-labeled anti TRA-1-81 (BD Biosciences), and APC-labeled anti SSEA4 (R&D systems) on ice in the dark for 1 hour. The cells were then washed and resuspended in cold PBS/BSA solution and analyzed within 30 minutes. Data was analyzed using FlowJo software (Treestar, Inc., San Carlos, CA).

**Quantitative real-time PCR.** Total RNA was extracted and purified using a PureLink^TM^ RNA Mini kit (Invitrogen, USA) per manufacturer recommendations.  PCR was carried out for 40 cycles and relative expression level for each target gene was evaluated using 2^-ΔΔCt^ method. To obtain the ΔCt values, the Ct values of target genes were normalized by the Ct values of GAPDH.

**Supplementary figures**


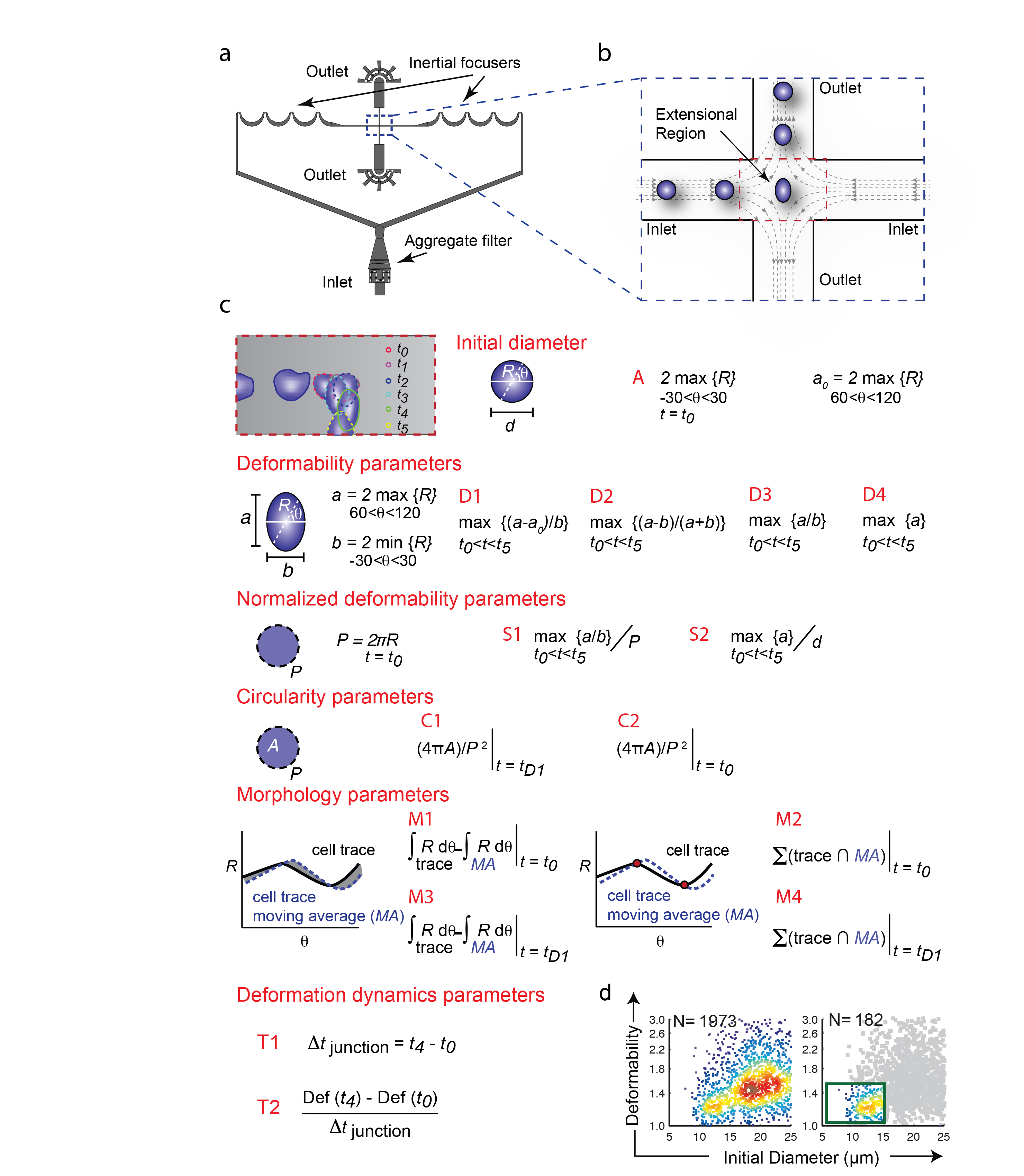


**Supplementary Figure 1:** Deformability cytometry device. Device schematic (a) shows the aggregate filter at the inlet, the curved channels facilitating inertial focusing and the extensional region magnified in b. (b) The extensional region is continuously imaged by high-speed camera. (c) A series of overlapped images show how a cell deforms as it enters the extensional region. Several parameters including initial diameter, deformability*,* circularity, morphology, stretching period and strain rate are captured for each cell using image processing. (d) The deformability and cell size parameters captured from cells are depicted as color density plots. Gating can further be used to identify specific cell populations.

**
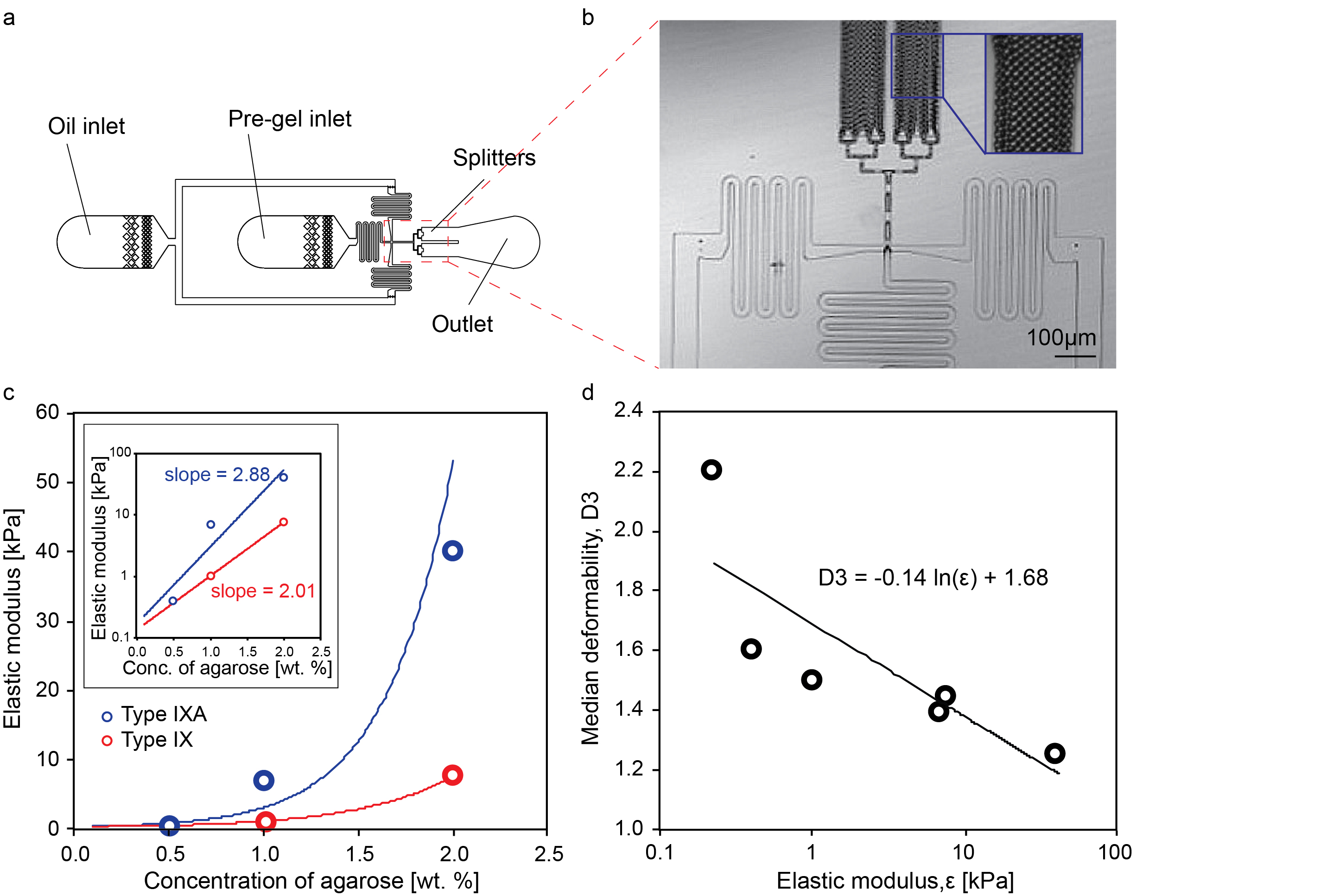
**

**Supplementary Figure 2:** Agarose calibration particles. (a) A microfluidic droplet-generating platform was used to generate agarose gel particles with different stiffness. (b) High-speed image showing the generation of agarose droplets sheathed in an oil/surfactant solution. Droplets are subsequently gelled upon refrigeration. (c) The elastic modulus of low-gelling temperature agarose gels with varying weight % of agarose were measured in bulk using AFM. (d) Using DC measurements, the median deformability of beads are plotted versus their mechanical stiffness. For these experiments gel diameters were fixed at 12±3 µm. This calibration curve can be used to translate deformability measurements to conventional cell stiffness measurements.


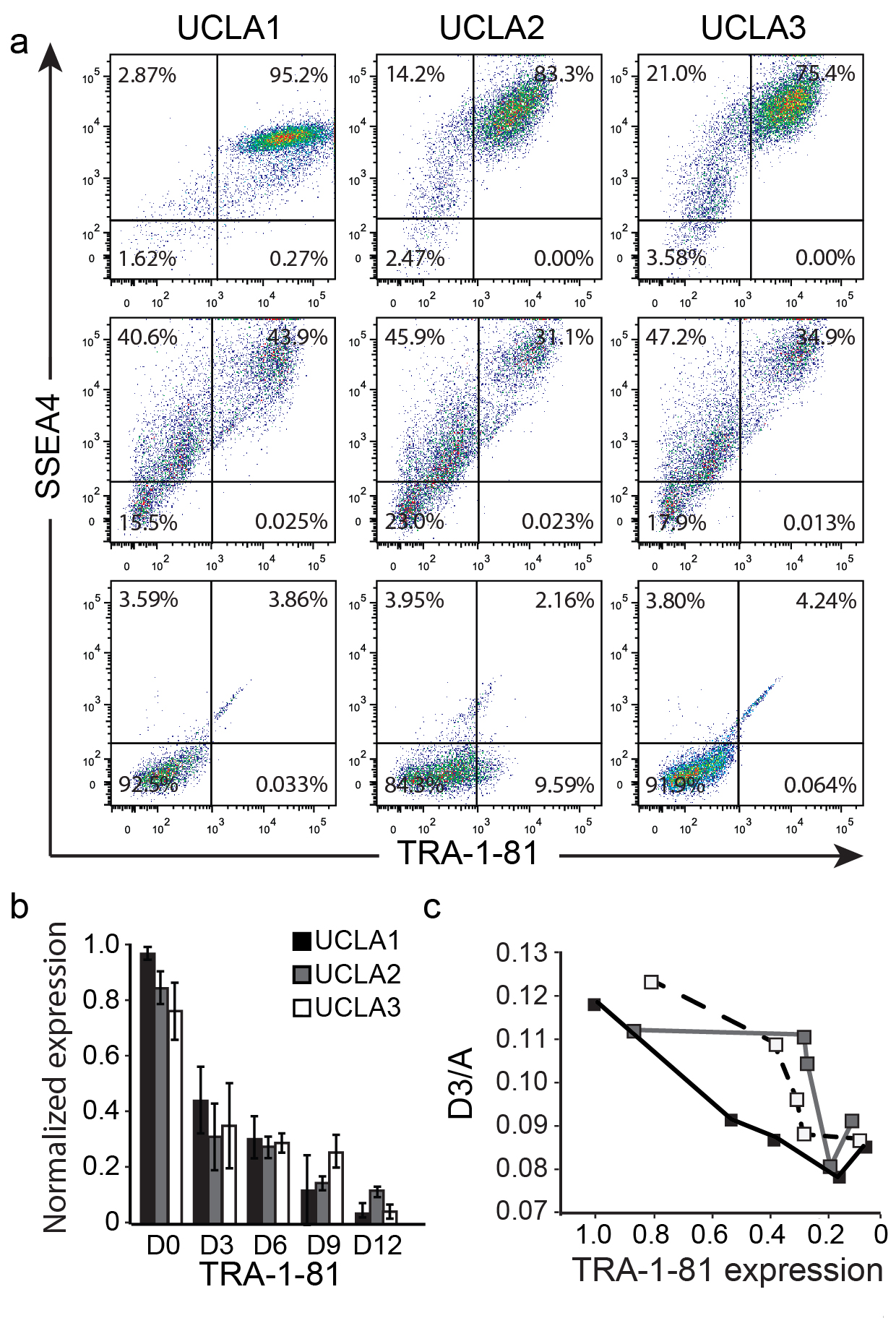


**Supplementary Figure 3:** (a) Down-regulation of SSEA4 and TRA-1-81 pluripotency markers was observed by flow cytometry for UCLA1-3 at day0 (first row), day3 (second row) and day12 (third row). (b) The largest change is seen within the first 3 days, but levels continue to decrease up to 12 days. (c) Correlation is observed between the expression of these conventional pluripotency measures and normalized deformability.


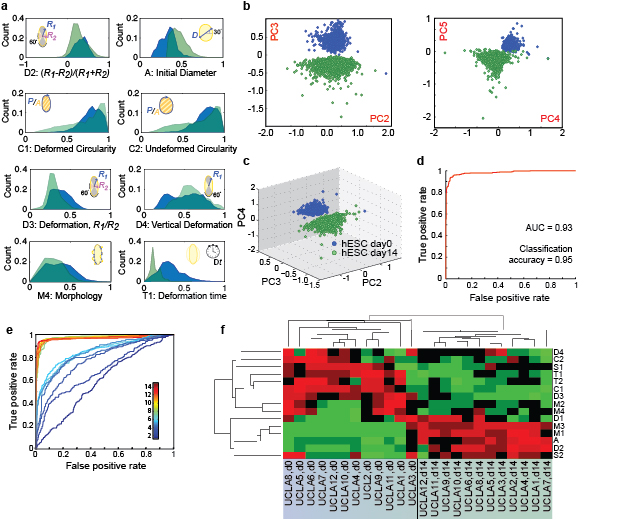


**Supplementary Figure 4:**

Multiparameter screening of mechanical and morphological properties can classify single cells accurately based on their pluripotency. (a) Histograms (normalized count) showing the variations within cell populations are depicted for several parameters showing overlap at the single-cell level for single properties (day0 in blue and day14 in green). (b,c) Labeling the data points as day0 (blue) and day14 (green) samples in principle component space shows distinct clusters corresponding to the two cell states. Note larger variation is present within the day14 cluster compared to day0. (d) ROC graph showing the performance of a linear kernel SVM classifier with all 15 parameters. (e) Using RFE and removing parameters one by one, ROC curves for different numbers of parameters show a decline in classifier performance with smaller numbers of parameters. (f) Linear Discriminant Analysis (LDA) successfully grouped pluripotent cells (day0) separately from differentiated cells (day14). LDA analysis also yielded the most important parameters contributing to the classification. Based on LDA the five most important parameters include deformability (D3), normalized deformability (S1,S2), size (A) and morphology (M1) (SI Table 1).

**
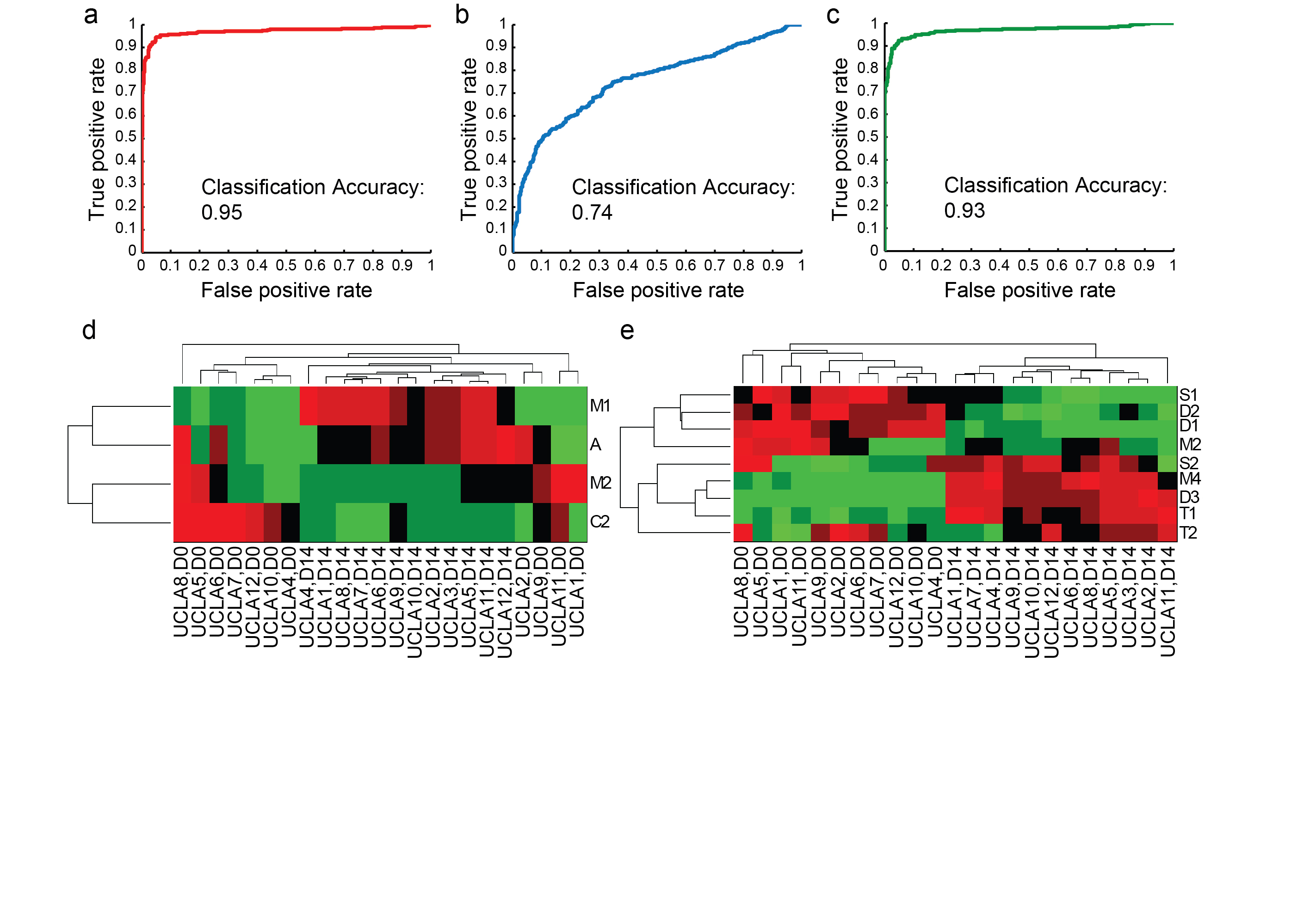
**

**Supplementary Figure 5:** Dynamic mechanical properties of cells are critical for accurate classification. (a) ROC plot showing the performance of the SVM classifier using all 15 biophysical parameters. (b) Using only the morphology parameters captured before the junction, classification accuracy dropped substantially to 74%. (c) Only considering parameters captured at the junction (during cell deformation) we could classify cells based on their pluripotency with 93% accuracy. (d,e) Clustering of cell populations using only pre-junction (d) and in-junction (e) parameters respectively. Four day0 samples were misclassified when only pre-junction parameters were used.

**
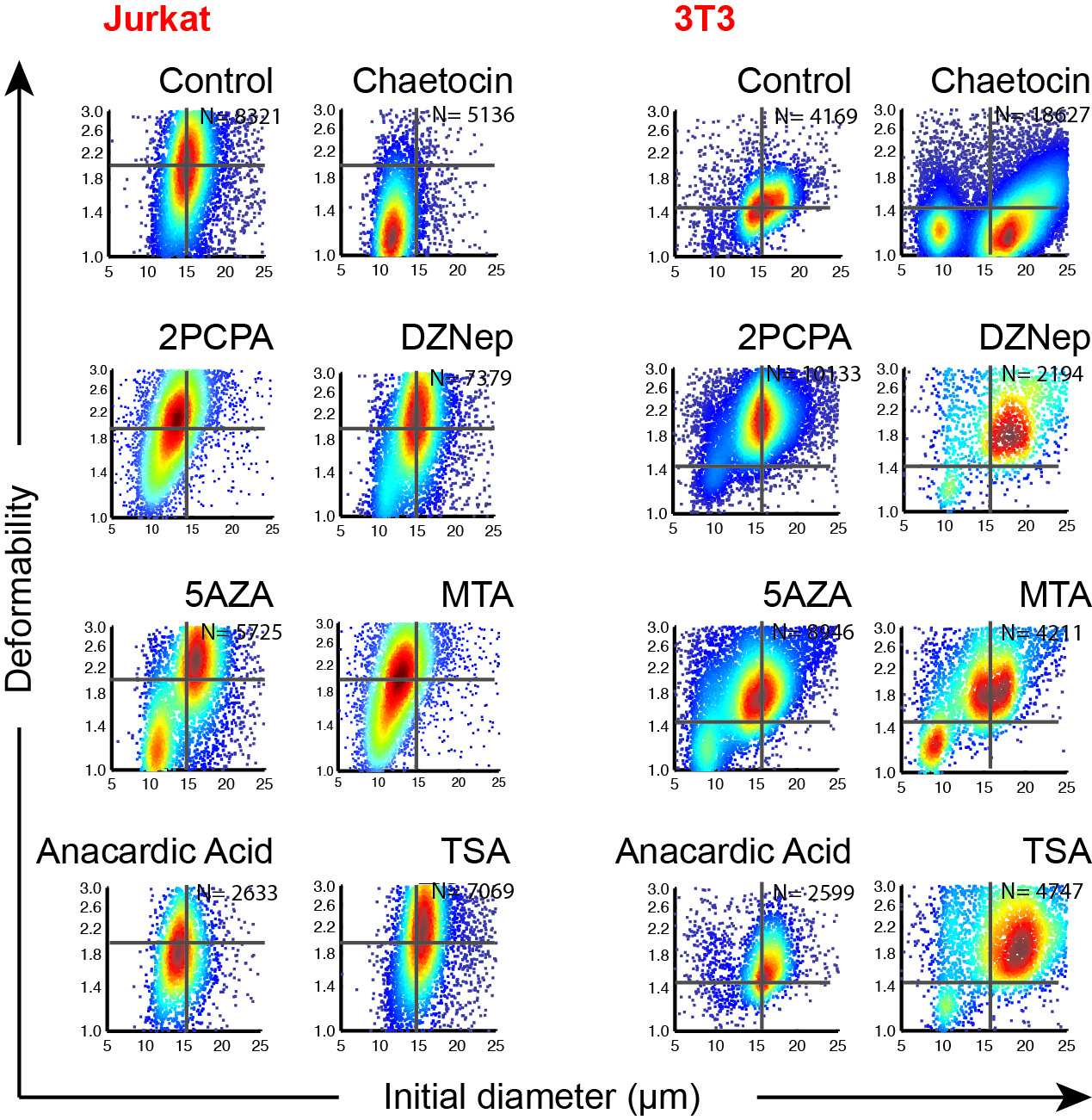
**

**Supplementary Figure 6:** Jurkat acute T-cell leukemia cells (left) and NIH-3T3 fibroblasts (right) were treated with different chromatin reorganizing drugs. Compared to control, treatment with H3K9me inhibitor, chaetocin and HAT inhibitor, anacardic acid, resulted in stiffening of cells, while treatment with HDAC inhibitor TSA, HMT inhibitor DZNep and DNMT inhibitors MTA and 5AZA resulted in an increase in cell deformability.

**
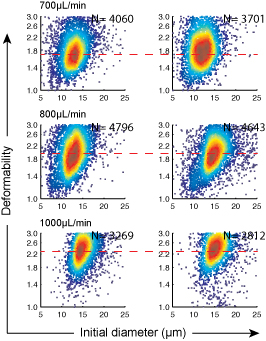
**

**Supplementary Figure 7:** No significant difference in the deformability of DNMT triple knockout mouse embryonic stem cells (right column) was observed compared to wildtype cells (left column) at three different flow rates: 700, 800 and 1000 µL/min.

**
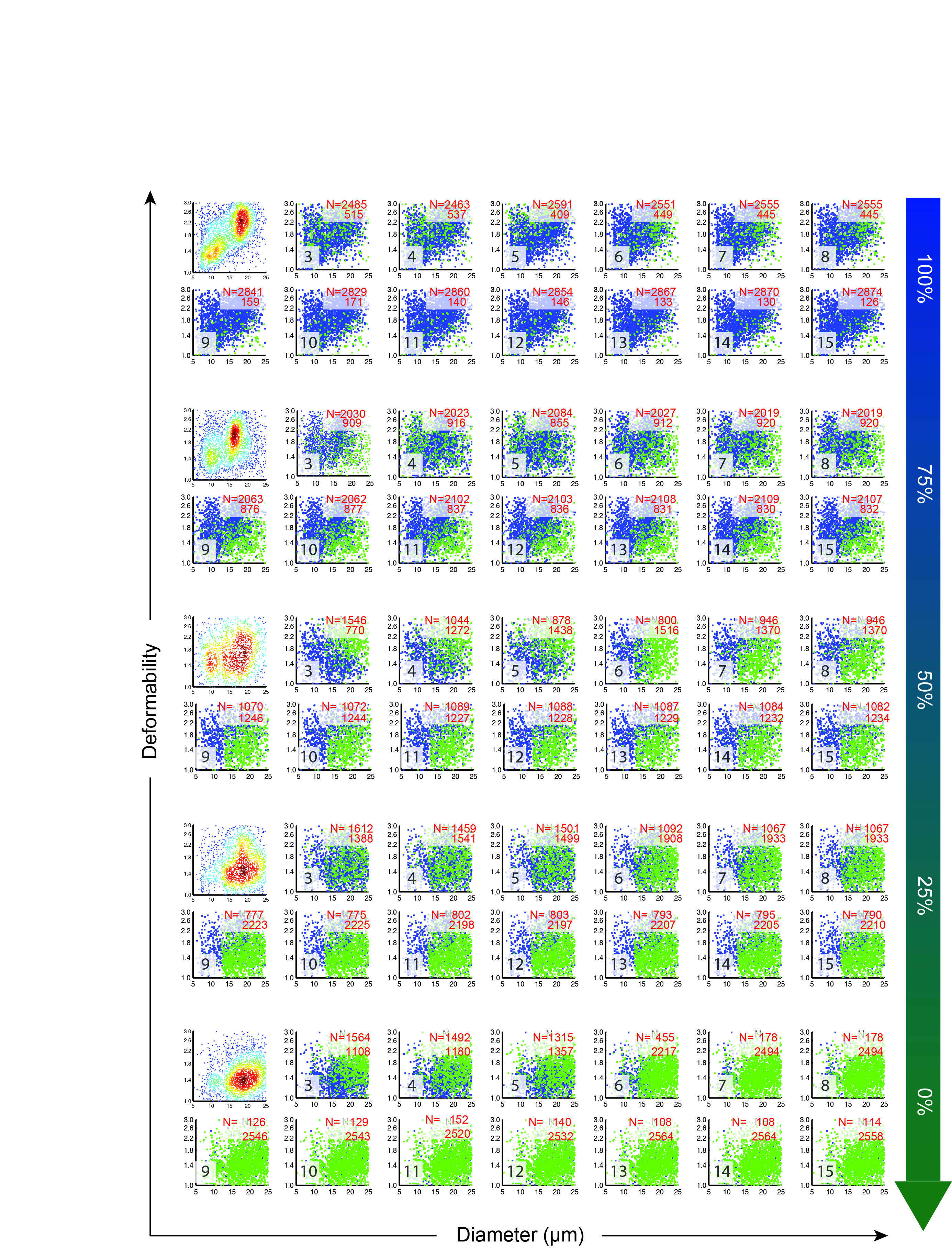
**

**Supplementary Figure 8:** Spiked sample classification with SVM for increasing numbers of parameters. For each spiked sample (containing 100, 75, 50, 25 or 0 percent day0 cells) data points classified as day0 (blue, number shown in top right insets - top) and day14 (green, number shown in top right insets - bottom) are shown using 3 to 15 parameters (shown in bottom left insets). The data is depicted in the two dimensional space of deformability and cell diameter.


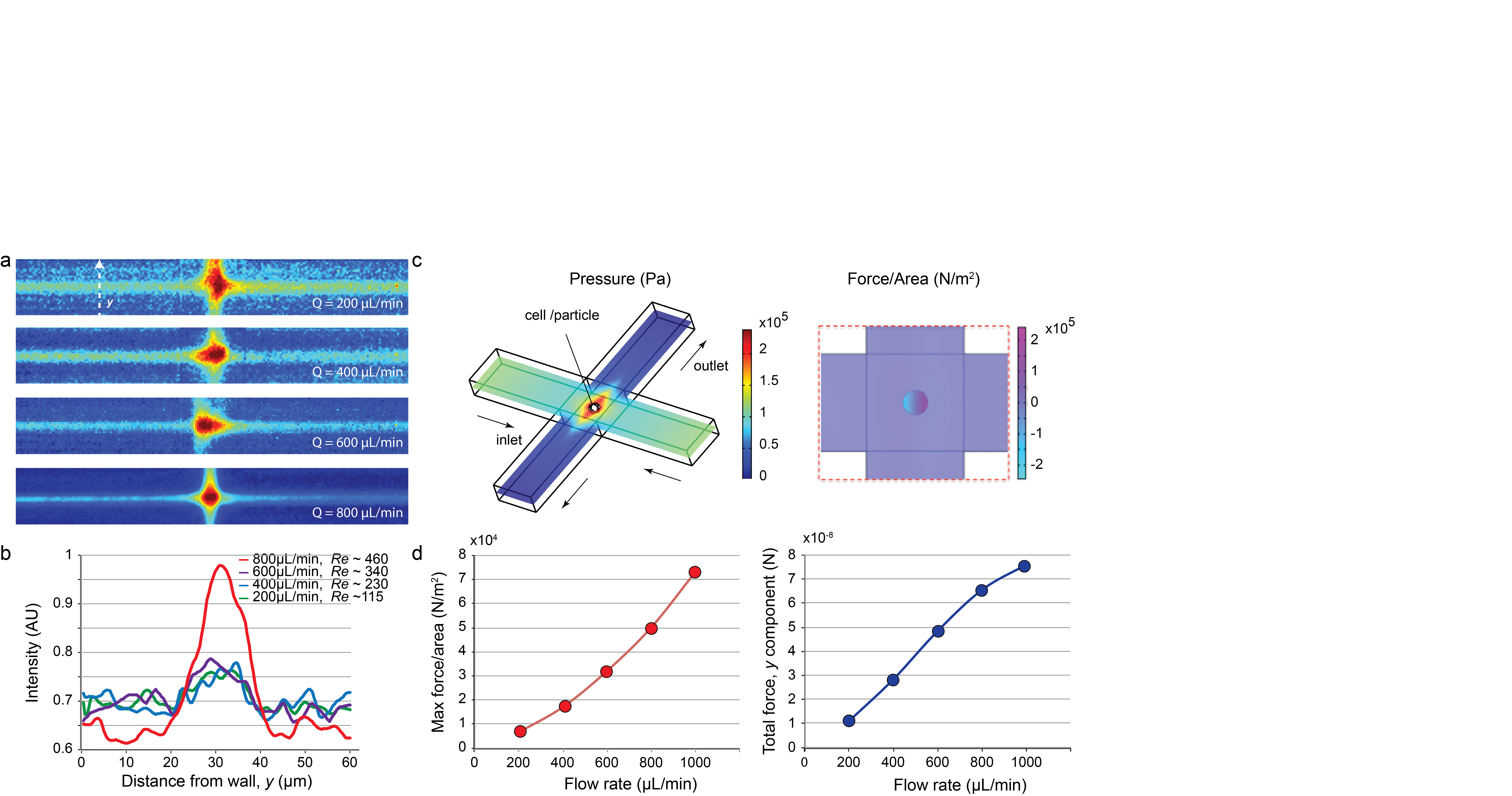


**Supplementary Figure 9:** Applying uniform force on cells (a) Flow rate should be high enough to focus cells at a single lateral position in order to apply uniform force to cells of the same size. Streak images show trajectories and junctional stretching uniformity as a function of flow rate. (b) Standard deviation plots showing the variations in lateral position of cells arriving at the junction and optimal operation at 800 µL/min. (c) Comsol simulations predict that the pressure on the cell is around 10^5^ Pa at the extensional region when flow rate is ~1000 µL/min. The model assumes a stationary cell. (d) Maximum force/area and the total y component of force on cells in the junction increase with increasing operating flow rate.

**
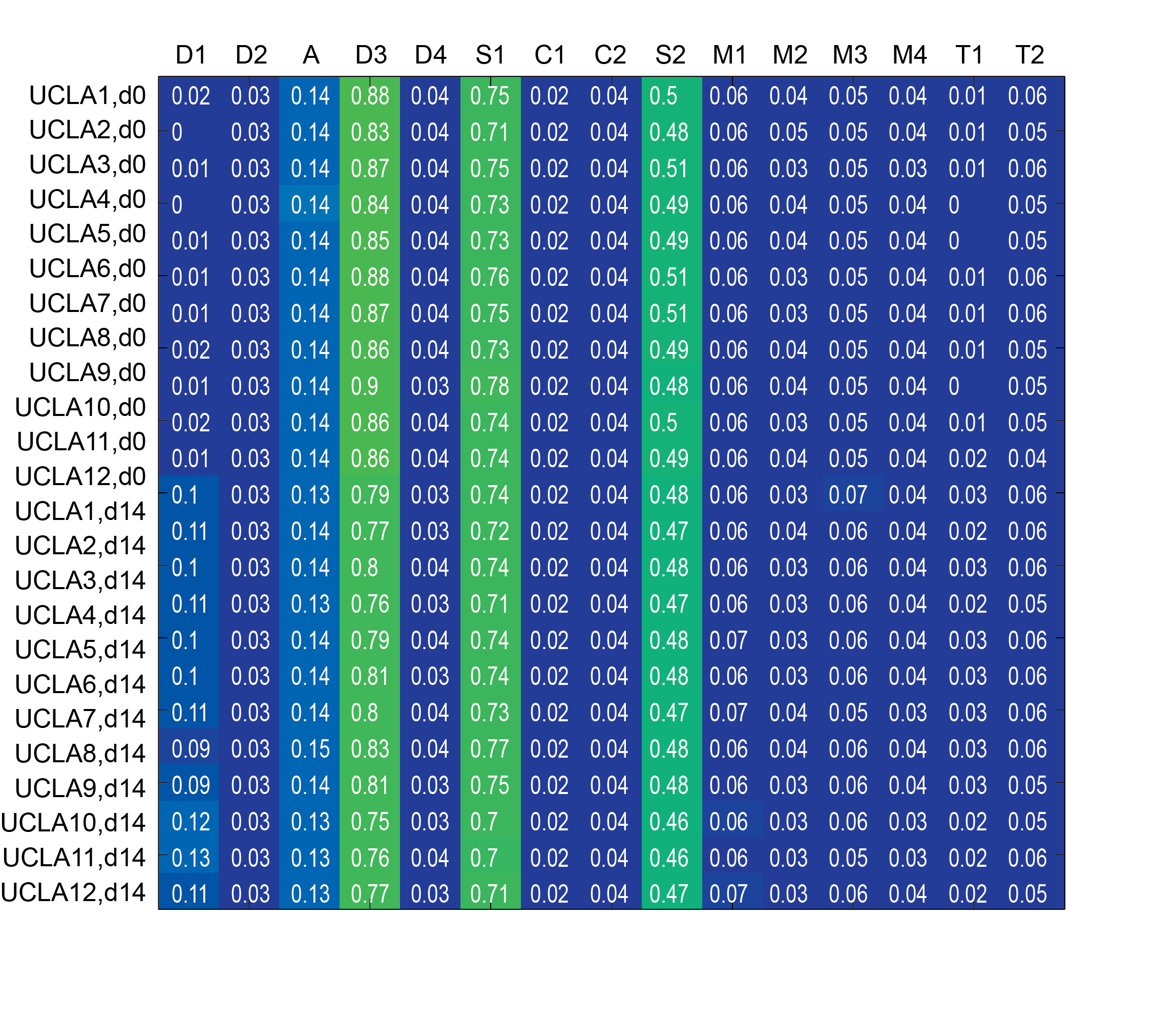
**

**Supplementary Table 1.** Linear discriminant analysis (LDA) coefficients for classifying 12 hESC lines before and after 2 weeks of differentiation. The 6 parameters with maximum average coefficients are deformability (D3), relative strain parameters (S1,S2) cell diameter (A), morphology (M1) and deformation time (T2)

**
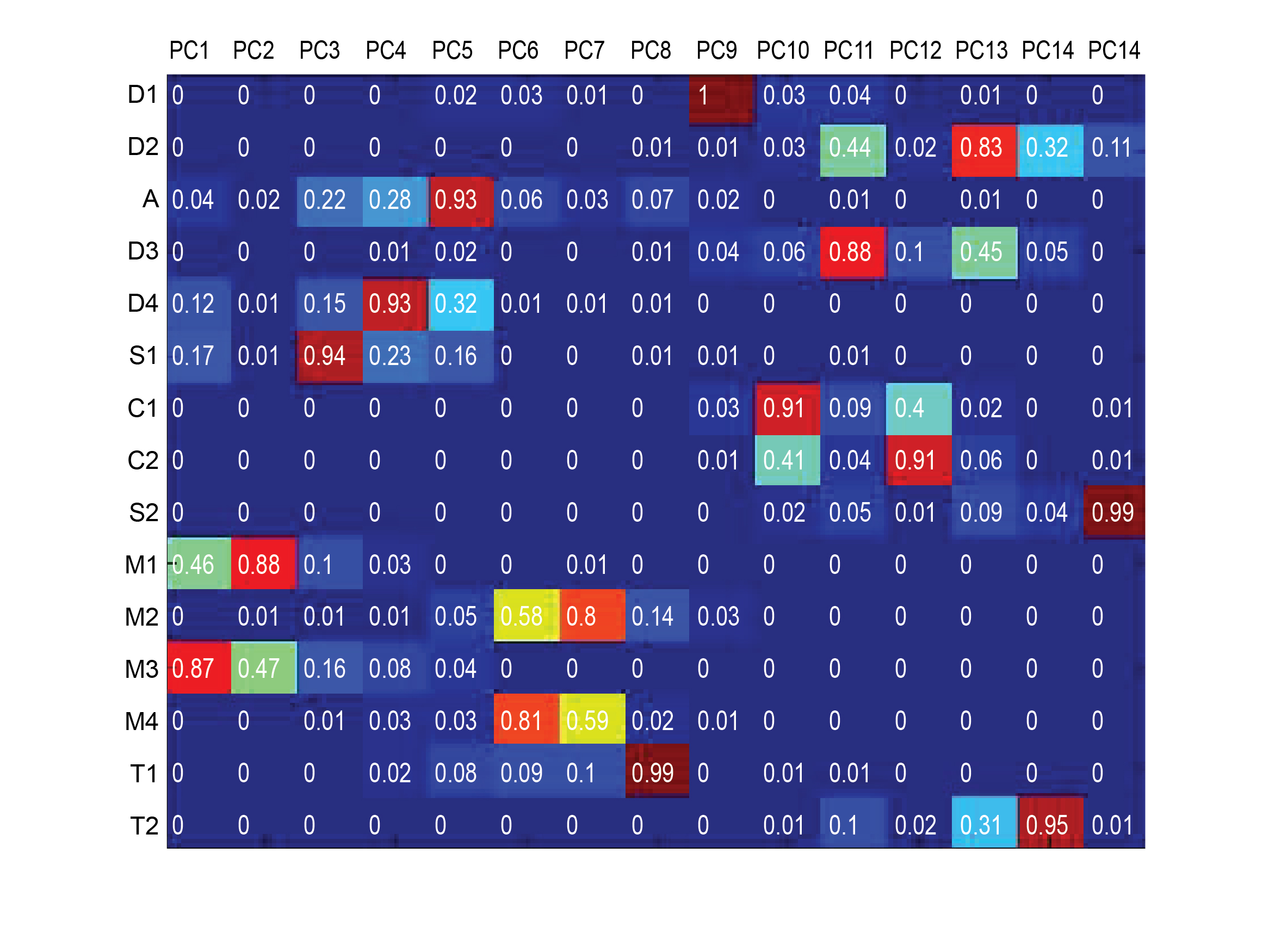
**

**Supplementary Table 2.** PCA components show a strong dependence of the most important PCs, PC1, PC2 and PC3 on parameters S1, M1, M3, A and D4 (absolute values of coefficients are shown).

**
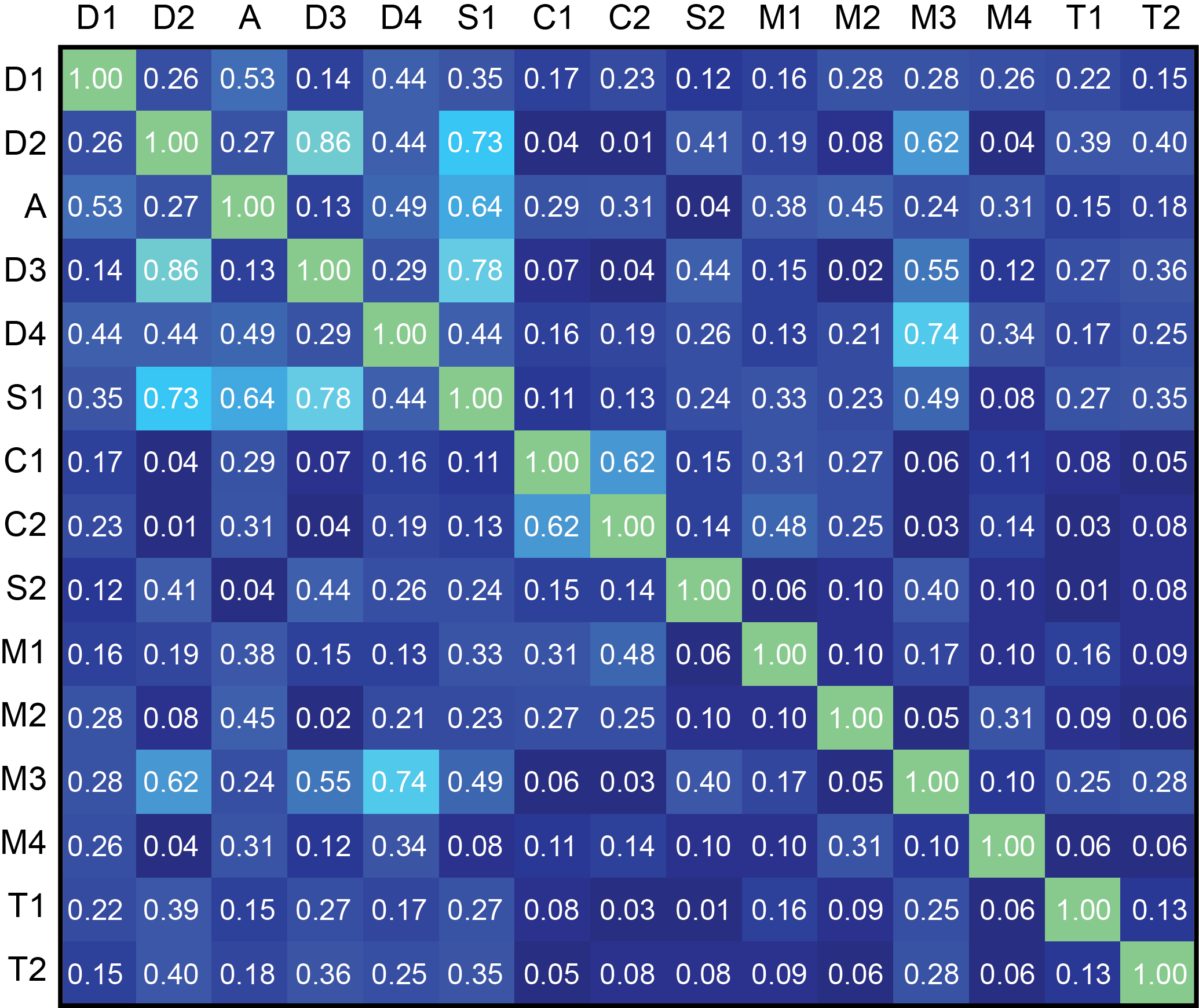
**

**Supplementary Table 3.** Correlation coefficient matrix (absolute coefficient values) shows some level of linear dependence between several parameters. The highest correlation was observed between parameters D2 and D3, which are both deformability measures at the junction as well as D3 and S1, which are deformability and normalized deformability measures at the junction. The least correlation is observed between S2 and T1.

**Supplementary videos**

SI video 1: Human embryonic stem cells (day0) deforming at the extensional flow region.

SI video 2: Fourteen-day differentiated human embryonic stem cells (day14) deforming at the extensional flow region.

SI video 3: A collection of cells deforming at the junction and their transformation from polar to Cartesian coordinate system. The white lines show the edges of the cells detected by the Matlab image processing script. The 15 parameters are extracted from the Cartesian maps as described in SI Fig.1.

SI video 4: Showing the polar to Cartesian coordinate system transformation of a sequence of images captured from one cell deforming in the device.

References:

1. Sullivan, T. *et al.* Loss of a-Type Lamin Expression Compromises Nuclear Envelope Integrity Leading to Muscular Dystrophy. *J. Cell Biol.* **147,** 913–920 (1999).

2. Coffinier, C. *et al.* Deficiencies in lamin B1 and lamin B2 cause neurodevelopmental defects and distinct nuclear shape abnormalities in neurons. *Mol. Biol. Cell* **22,** 4683–4693 (2011).

3. Jung, H.-J. *et al.* Farnesylation of lamin B1 is important for retention of nuclear chromatin during neuronal migration. *Proc. Natl. Acad. Sci.* **110,** E1923–E1932 (2013).

4. Tsumura, A. *et al.* Maintenance of self‐renewal ability of mouse embryonic stem cells in the absence of DNA methyltransferases Dnmt1, Dnmt3a and Dnmt3b. *Genes Cells* **11,** 805–814 (2006).

5. Gossett, D. R. *et al.* Hydrodynamic stretching of single cells for large population mechanical phenotyping. *Proc. Natl. Acad. Sci.* **109,** 7630–5 (2012).

6. Eun, Y.-J., Utada, A. S., Copeland, M. F., Takeuchi, S. & Weibel, D. B. Encapsulating bacteria in agarose microparticles using microfluidics for high-throughput cell analysis and isolation. *ACS Chem. Biol.* **6,** 260–266 (2010).
